# C5a Induces the Synthesis of IL-6 and TNF-α in Rat Glomerular Mesangial Cells through MAPK Signaling Pathways

**DOI:** 10.1371/journal.pone.0161867

**Published:** 2016-09-01

**Authors:** Mingde Ji, Yanlai Lu, Chenhui Zhao, Wenxing Gao, Fengxia He, Jing Zhang, Dan Zhao, Wen Qiu, Yingwei Wang

**Affiliations:** 1 Department of Immunology, Nanjing Medical University, Nanjing, Jiangsu, 211166, P.R. China; 2 Department of Laboratory Medicine, Affiliated Hospital of Nanjing University of Traditional Chinese Medicine, Nanjing, Jiangsu, 210029, P.R. China; 3 Department of Medicine, First Affiliated Hospital of Nanjing Medical University, Nanjing, Jiangsu, 210029, P.R. China; 4 Basic Medical Science of Basic Medical College, Nanjing Medical University, Nanjing, Jiangsu, 211166, P.R. China; Toho Daigaku, JAPAN

## Abstract

Inflammatory response has been reported to contribute to the renal lesions in rat Thy-1 nephritis (Thy-1N) as an animal model of human mesangioproliferative glomerulonephritis (MsPGN). Besides C5b-9 complex, C5a is also a potent pro-inflammatory mediator and correlated to severity of various nephritic diseases. However, the role of C5a in mediating pro-inflammatory cytokine production in rats with Thy-1N is poorly defined. In the present studies, the levels of C5a, interleukin-6 (IL-6) and tumor necrosis factor-α (TNF-α) were first determined in the renal tissues of rats with Thy-1N. Then, the expression of IL-6 and TNF-α was detected in rat glomerular mesangial cells (GMC) stimulated with our recombinant rat C5a *in vitro*. Subsequently, the activation of mitogen-activated protein kinase (MAPK) signaling pathways (p38 MAPK, ERK1/2 and JNK) and their roles in the regulation of IL-6 and TNF-α production were examined in the GMC induced by C5a. The results showed that the levels of C5a, IL-6 and TNF-α were markedly increased in the renal tissues of Thy-1N rats. Rat C5a stimulation *in vitro* could up-regulate the expression of IL-6 and TNF-α in rat GMC, and the activation of MAPK signaling pathways was involved in the induction of IL-6 and TNF-α. Mechanically, p38 MAPK activation promoted IL-6 production, while either ERK1/2 or JNK activation promoted TNF-α production in the GMC with exposure to C5a. Taken together, these data implicate that C5a induces the synthesis of IL-6 and TNF-α in rat GMC through the activation of MAPK signaling pathways.

## Introduction

Human mesangioproliferative glomerulonephritis (MsPGN) is a renal disease characterized by glomerular mesangial cell (GMC) apoptosis and proliferation as well as extracellular matrix (ECM) secretion [1–4]. Recently, inflammatory response has been considered to be an important contributor to the development of MsPGN [5–7]. Rat Thy-1 nephritis (Thy-1N) is a widely used animal model for studying human MsPGN [8–11]. Multiple evidences support that similar to human MsPGN, GMC of Thy-1N rats undergo complex pathological changes including inflammation, apoptosis and proliferation [12–18]. Nevertheless, the precise role of inflammatory response especially pro-inflammatory mediator or cytokine production in rat Thy-1N and its regulatory mechanism are largely unclear.

During the induction of rat Thy-1N, the Thy-1 Ab binds to the Thy-1 antigen on GMC membrane and forms immune complex, the later activates the classical pathway of complement system to generate C5b-9 complex and other activated fragments such as C5a. It has been revealed that C5b-9 especially sublytic C5b-9 can trigger a series of biochemical events and lead to cell inflammation, apoptosis and proliferation in rat Thy-1N [16, 18, 19]. In addition, C5a has broad pro-inflammatory activity through its receptor C5aR, and contributes to the pathogenesis of some types of nephritis such as anti-glomerular basement (GBM) glomerulonephritis, lupus nephritis, anti-myeloperoxidase (MPO)-induced necrotizing crescentic glomerulonephritis (NCGN) [20–23]. However, the effects of C5a on the induction of pro-inflammatory cytokines in the GMC of Thy-1N rats have not been elucidated.

It has been reported that pro-inflammatory cytokines including interleukin-6 (IL-6) and tumor necrosis factor-α (TNF-α) are markedly up-regulated in the renal tissues of rats with Thy-1N [18, 24]. Additionally, our present studies revealed that the renal C5a content was significantly increased in rat Thy-1N, and its production was earlier than IL-6 and TNF-α production. Given that C5a is also able to promote the synthesis of pro-inflammatory cytokines in some types of cells [25, 26], whether C5a can induce the production of IL-6 and TNF-α in the GMC needs to be clarified.

In the present study, the mRNA and/or protein levels of C5a, IL-6 and TNF-α were first determined in the renal tissues of rats with Thy-1N. Then, recombinant rat C5a protein was prepared via prokaryotic expression, and its nucleotide sequence, protein molecular weight and biological activity were identified. Subsequently, the expression of IL-6 and TNF-α at mRNA and protein levels was detected in the GMC stimulated with the rat C5a *in vitro*. In addition, as for the upstream regulation of IL-6 and TNF-α production, the activation of mitogen-activated protein kinase (MAPK) signaling pathways (p38 MAPK, ERK1/2 and JNK) was examined in the GMC induced by C5a.

## Materials and Methods

### Reagents

Rabbit polyclonal antibody against C5a (250565) was from Abbiotec (San Diego, CA, USA). Monoclonal antibodies against C5aR (sc-25774) and His-tag (sc-53073) were supplied by Santa Cruz Biotechnology (Dallas, TX, USA). Rabbit monoclonal antibodies against p38 MAPK (9212), phos-p38 MAPK (p-p38 MAPK, 4511), ERK1/2 (4695), phospho-ERK1/2 (p-ERK1/2, 4370), JNK (9258), phospho-JNK (p-JNK, 4668) were purchased from Cell Signaling Technology (Danvers, MA, USA). Mouse monoclonal antibody against β-actin (BM0627) was supplied by Boster (Wuhan, China). IRDye 800CW-conjugated anti-mouse IgG (926–32210) was from LI-COR (Lincoln, NE, USA). Horseradish peroxidase (HRP)-conjugated anti-rabbit IgG (7074) and HRP-conjugated anti-mouse IgG (7076) were purchased from Cell Signaling Technology. Enhanced chemiluminescence (ECL) substrate and RIPA lysis buffer were supplied by Thermo Fisher Scientific (Waltham, MA, USA). Trizol was purchased from Invitrogen (Carlsbad, CA, USA). A RevertAid^TM^ First Strand cDNA Synthesis Kit was supplied by Fermentas (Pittsburgh, PA, USA). Primers were synthesized by Invitrogen (shanghai, china). The plasmid of pET-21a and BL21 (DE3) Singles™ Competent Cells were obtained from Novagen (Madison, WI, USA). T4 DNA Ligase and restriction enzymes of *Nde* I and *Xho* I were from NEB (Ipswich, UK). B-PER^®^ Bacterial Protein Extraction Reagent was supplied by Thermo Fisher Scientific. High Affinity Ni-NTA Resin was purchased from Genscript (Nanjing, China). Escherichia coli endotoxin and dichlorofluorescein diacetate (DCFH-DA) were from Sigma-Aldrich (St. Louis, MO, USA). Transwell chamber was obtained from Corning (Corning City, New York, USA). The Rat C5a ELISA kit was purchased from Novateinbio (Woburn, MA, USA). The p38 MAPK inhibitor (SB203580), ERK1/2 inhibitor (U0126) and JNK inhibitor (SP600125) were obtained from Beyotime (Nantong, China). A kinetic turbidimetric Tachypleus Amebocyte Lysate (TAL) kit was supplied by Chinese Horseshoe Crab Reagent Manufactory Co., Ltd. (Xiamen, China).

### Thy-1N model induction and experimental design

Anti-thymocyte serum (ATS) containing rabbit polyclonal antibodies against rat Thy-1 antigen (Thy-1 Ab, titer 1:640) were prepared according to previously published procedures [27]. Male SD rats (180-200g) were from the Nanjing Medical University Laboratory Animal Center (Nanjing, China). All animal experiments were performed in compliance with the guide for the care and use of laboratory animals and were approved by the Institutional Animal Care and Use Ethics Committee of Nanjing Medical University. Rat Thy-1N was induced by administration of ATS namely Thy-1 Ab (0.75ml/100g) through a single i.p. injection [28]. Rat renal cortexes were obtained by sacrifice at different time points, and then examined for the protein levels of C5a, IL-6 and TNF-α by Western blot and ELISA assay respectively.

### Construction of pET-21a/His-C5a plasmid

Total RNA was extracted from the SD rat liver tissues with Trizol reagent. The first strand cDNA was then synthesized from total RNA using the RevertAidTM First Strand cDNA Synthesis Kit. Subsequently, the complete open reading frame (ORF) of rat C5a gene was amplified by polymerase chain reaction (PCR). At the same time, His-tag was conjugated to the 5’-end of C5a ORF. Specific primer sequences were as follows: forward primer, 5'-CGGAATTCCGATGCATCATCATCATCATCATGACCTGCAGCTCCTGC-3'; and the reverse primer, 5'-CGGGATCCCGTTACCTTCCCAACAGCATGCCTTTG-3'. The expression plasmid of pET-21a/His-C5a was then constructed by inserting the amplified rat C5a gene with His-tag-encoding sequence into pET-21a through restriction enzyme sites of *Nde* I and *Xho* I. Furthermore, the recombinant plasmid of pET-21a/His-C5a was amplified in Escherichia coli strain of DH5α. The constructed plasmid was sequenced across both junctions to confirm the nucleotide sequence.

### Prokaryotic expression, purification and identification of rat C5a

The plasmid of pET-21a/His-C5a was transformed into BL21 (DE3) Singles™ Competent Cells for protein expression and purification according to the manufacturer's instructions. The protein was extracted by B-PER^®^ Bacterial Protein Extraction Reagent from 50 mL bacteria according to the protocol. Then, High Affinity Ni-NTA Resin was used for His-tag purification of the recombinant rat C5a protein according to the manufacturer's instructions. Subsequently, the molecular weight of the recombinant rat C5a protein was identified by Western blot. Additionally, the quantification of endotoxin (EU/ml) in the purified C5a was determined using a commercial kinetic turbidimetric TAL kit according to the manufacturer’s instruction [29].

### Neutrophils reactive oxygen species (ROS) assay

Rat polymorphonuclear neutrophils (PMN) were separated with the plasma-Percoll according to the Haslett’s method [30]. PMN were stimulated with the recombinant rat C5a at the dose of 50 ng/ml in PBS at 37°C for 5 min as previously described [31, 32], and then incubated with DCFH-DA (10 μM) in PBS at 37°C for 30 min. Subsequently, ROS generation in PMN was detected on a FACSCanto II flow cytometer (BD, Franklin Lakes, NJ, USA). PMN activation rate = DCFH-positive cells / total PMN cells × 100%.

### Neutrophil chemotaxis assay

1.0 ml of serum-free MEM with the recombinant rat C5a (50 ng/ml) was added to the lower chamber of the transwell system, 300 μl of serum-free MEM with neutrophils was transferred to the upper chamber of the transwell system, and then incubated at 37°C for 1 h. Next, the upper chamber was removed, and the liquid in the upper chamber was siphoned off with cotton swab. The cells on the membrane were wiped with cotton swab. After drying naturally, the membrane was stained with 0.1% crystal violet for 10 min and was washed twice with PBS. The neutrophils on the membrane were observed under the microscope.

### GMC culture and C5a stimulation

Rat GMC cell line was provided by China Centre for Type Culture Collection (Wuhan, China). Rat GMC were cultured and maintained in modified Eagle’s medium (MEM) supplemented with 10% Fetal bovine serum (FBS), 100 mg/ml penicillin and 100 mg/ml streptomycin, and used for experiments [19]. Rat GMC were incubated with the recombinant rat C5a protein at the dose of 50 ng/ml for further detection of IL-6 and TNF-α production and MAPK signaling pathway activation.

### C5aR siRNA generation and transfection

To silence rat C5aR gene, three different siRNA sequences against rat C5aR mRNA (NM053619.1) were designed and synthesized by GenePharma (Shanghai, China). Subsequently, the three C5aR siRNAs were transfected into rat GMC respectively by using Neon^TM^ transfection system as described before [16, 19], and the most effective C5aR siRNA (5’-CGCUCAUUCUGCUCAACAUTT-3’) was chosen for further experiments. At the same time, the control siRNA was produced as a negative control.

### Reverse transcription-PCR (RT-PCR)

Total RNA was extracted from the rat renal tissues and the cultured rat GMC with different treatment by TRIzol reagent. A total of 1 μg RNA was then used for the first-strand cDNA synthesis by the RevertAid^TM^ First Strand cDNA Synthesis Kit according to the manufacture’s protocols. The primers used for PCR amplification were shown as follows: IL-6 primers forward, 5'-TTGCCTTCTTGGGACTG-3' and reverse, 5'-CTGGCTTTGTCTTTCTTGTTA-3'; TNF-α primers forward, 5'-GTCGTAGCAAACCACCAAG-3', and reverse, 5'-GTCGCCTCACAGAGCAAT-3'; GAPDH primers forward, 5'-ACCACAGTCCATGCCATCAC-3', and reverse, 5'-TCCACCACCCTGTTGCTGTA-3'. GAPDH expression in each sample was identified as the internal standard.

### Western blot

The proteins (40 μg / well) were subjected to 10–12% SDS polyacrylamide gel or 4–20% ExpressPlus PAGE Gel (Genscript, Nanjing, China) for electrophoresis and transferred onto PVDF membranes by PowerPac^TM^ Basic (Bio-Rad, Hercules, USA). The PVDF membranes were incubated in blocking buffer (5% skim milk in TBS-T buffer) at room temperature (RT) for 1 h and then incubated with the antibodies to C5a, C5aR, His-tag, p38 MAPK, p-p38 MAPK, ERK1/2, p-ERK1/2, JNK, p-JNK and β-actin at 4°C overnight. β-actin expression in each sample was identified as the internal standard. The phosphorylation levels of p38 MAPK, ERK1/2 and JNK were normalized to the total levels of p38 MAPK, ERK1/2 and JNK respectively. After 5 times of washing with TBST-T, the PVDF membranes were further incubated with IRDye 800CW-conjugated anti-mouse IgG, HRP-conjugated anti-rabbit IgG and HRP-conjugated anti-mouse IgG at RT for 1 h. The bands were visualized by Odyssey system (for the recombinant rat C5a identification) or regular X-ray film through ECL detection system after washing the PVDF membranes 5 times. Finally, the density of radiographic band onto PVDF membranes was analyzed by using the software of Quantity One (Bio-Rad).

### ELISA

The protein levels of IL-6 and TNF-α in the renal tissues of Thy-1N rats and in the supernatant of cultured rat GMC were determined using ELISA kits according to the manufactures’ instructions.

### Statistical analysis

Data are presented as mean ± SD. One-way ANOVA was used to determine significant differences among groups. Where significant differences were found, individual comparisons were made between groups using the Student's t test and adjusting the critical value according to the Bonferroni method. P < 0.05 was considered significant.

## Results

### C5a, IL-6 and TNF-α were increased in the renal tissues of rats with Thy-1N

**Fig 1:**
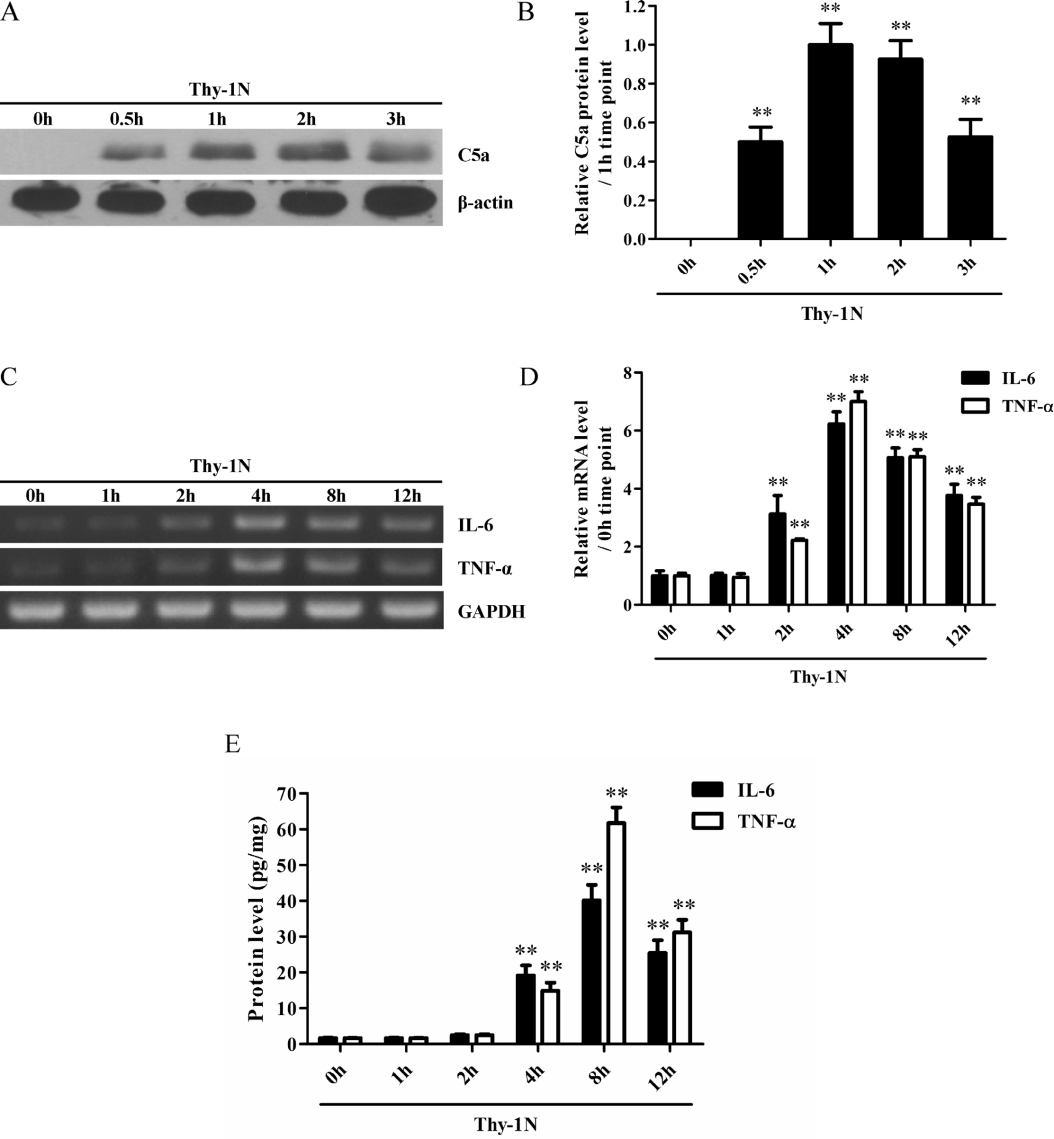
The levels of C5a, IL-6 and TNF-α protein in the renal tissues of rats with Thy-1N. (A and B) Rat Thy-1N was induced and then the protein level of C5a in the rat renal tissues was detected at different time points (0 h, 0.5 h, 1 h, 2 h and 3 h; *n* = 4 in each time point) after Thy-1N induction by Western blot assay. (C-E) The mRNA and protein levels of IL-6 and TNF-α in the renal tissues of Thy-1N rats was detected at various time points (0 h, 1 h, 2 h, 4 h, 8 h and 12 h; *n* = 6 in each time point) after Thy-1N induction by RT-PCR (C and D) and ELISA (E) respectively. Results were represented as means ± SD. Representative photographs were shown. ** *P*<0.01 *versus* 0 h time point (non-treated).

Rat Thy-1N was reproduced and then the mRNA and/or protein levels of C5a, IL-6 and TNF-α in the rat renal tissues were detected at different time points after the nephritis induction. The time course experiments revealed that the C5a protein production in the renal tissues of rats with Thy-1N was markedly increased with a maximum at 1 h (Fig 1A and 1B). The mRNA and protein levels of both IL-6 and TNF-α were obviously enhanced with a maximum at 4 h for mRNA expression and at 8 h for protein production respectively (Fig 1C–1E). There data indicates that C5a might contribute to the IL-6 and TNF-α induction and inflammatory lesions during rat Thy-1N. Therefore, further *in vitro* studies were designed to explore the potential role of C5a in mediating the synthesis of pro-inflammatory cytokines in rat GMC.

### Identification and biological activity analysis of the recombinant rat C5a

**Fig 2:**
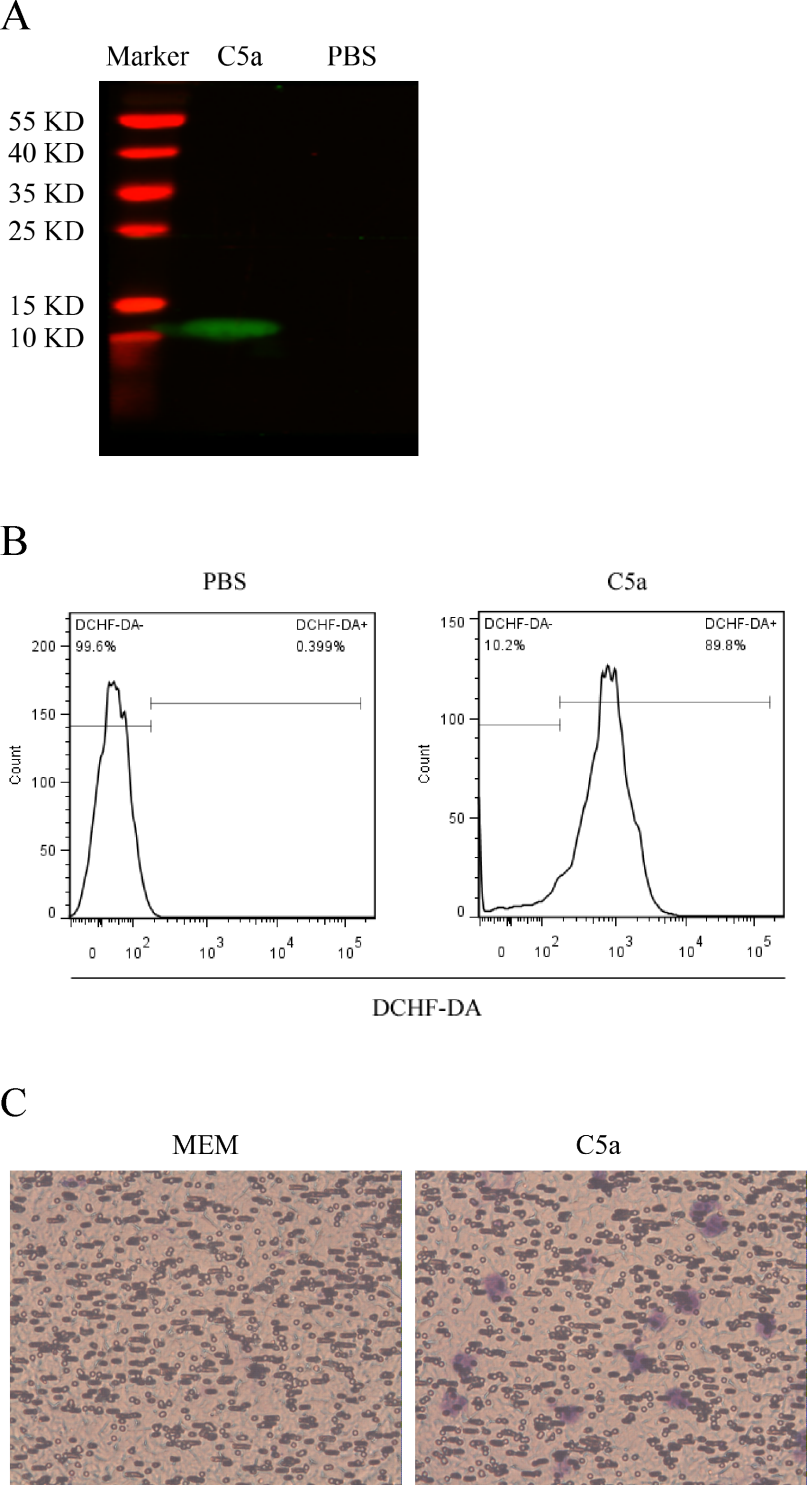
Recombinant rat C5a identification and its biological activity analysis. (A) Western blot was used to detect the recombinant rat His-C5a protein by the antibody to His-tag. (B) Neutrophils were stimulated with C5a (50 ng/ml) for 5 min, and then incubated with DCFH-DA (10 μM) for 30 min. Subsequently, flow cytometry was performed to detect the percentage of DCFH-DA-positive neutrophils. (C) Transwell assay was used to detect neutrophil chemotaxis induced by C5a stimulation at the dose of 50 ng/ml for 30 min. The neutrophils on the membrane were observed by crystal violet staining under the microscope. Representative photographs were manifested (*n* = 3 in each group).

Since there was no commercial rat C5a protein, in order to explore the role of C5a *in vitro*, we firstly produced recombinant rat C5a protein through prokaryotic expression. Briefly, the expression plasmid of pET-21a/His-C5a was constructed and sequenced to confirm the nucleotide sequence. Subsequently, prokaryotic expression in BL21 (DE3) Singles™ Competent Cells and His-tag-based purification through High Affinity Ni-NTA Resin were performed. Our recombinant rat C5a protein contained 83 amino acid residues including 6 amino acid residues of His-tag and 77 amino acid residues of rat C5a [33]. Based on above-mentioned sequence of the recombinant rat C5a protein, 9.81 KD of molecular weight was predicted by bio-soft (http://www.bio-soft.net/sms/prot_mw.html). Subsequently, the molecular weight of the recombinant rat C5a protein was identified by Western blot with anti-His-tag antibody, and a band of approximate 11 KD was found (Fig 2A). Notably, the difference of 1 KD was in the range of permitted errors, indicating that the molecular weight of the recombinant rat C5a protein is correct.

To identify the biological activity of recombinant rat C5a protein, both ROS production and chemotaxis activity were assayed in the neutrophils stimulated with rat C5a. DCFH-DA was first used to label ROS in the neutrophils after C5a stimulation for 5 min, and then flow cytometry was performed to detect the percentage of DCFH-DA-positive neutrophils. The results showed that C5a stimulation markedly increased ROS production in the neutrophils compared to PBS stimulation group (Fig 2B). Furthermore, neutrophil chemotactic experiment was done to determine the chemotaxis activity of the C5a, and the result exhibited that C5a stimulation for 30 min significantly induced the chemotaxis of neutrophils, but PBS treatment had no effect on the chemotaxis of neutrophils (Fig 2C). Taken together, these data demonstrated that we had successfully prepared recombinant rat C5a protein with biological activity.

### The expression of IL-6 and TNF-α was up-regulated in rat GMC stimulated with C5a

**Fig 3:**
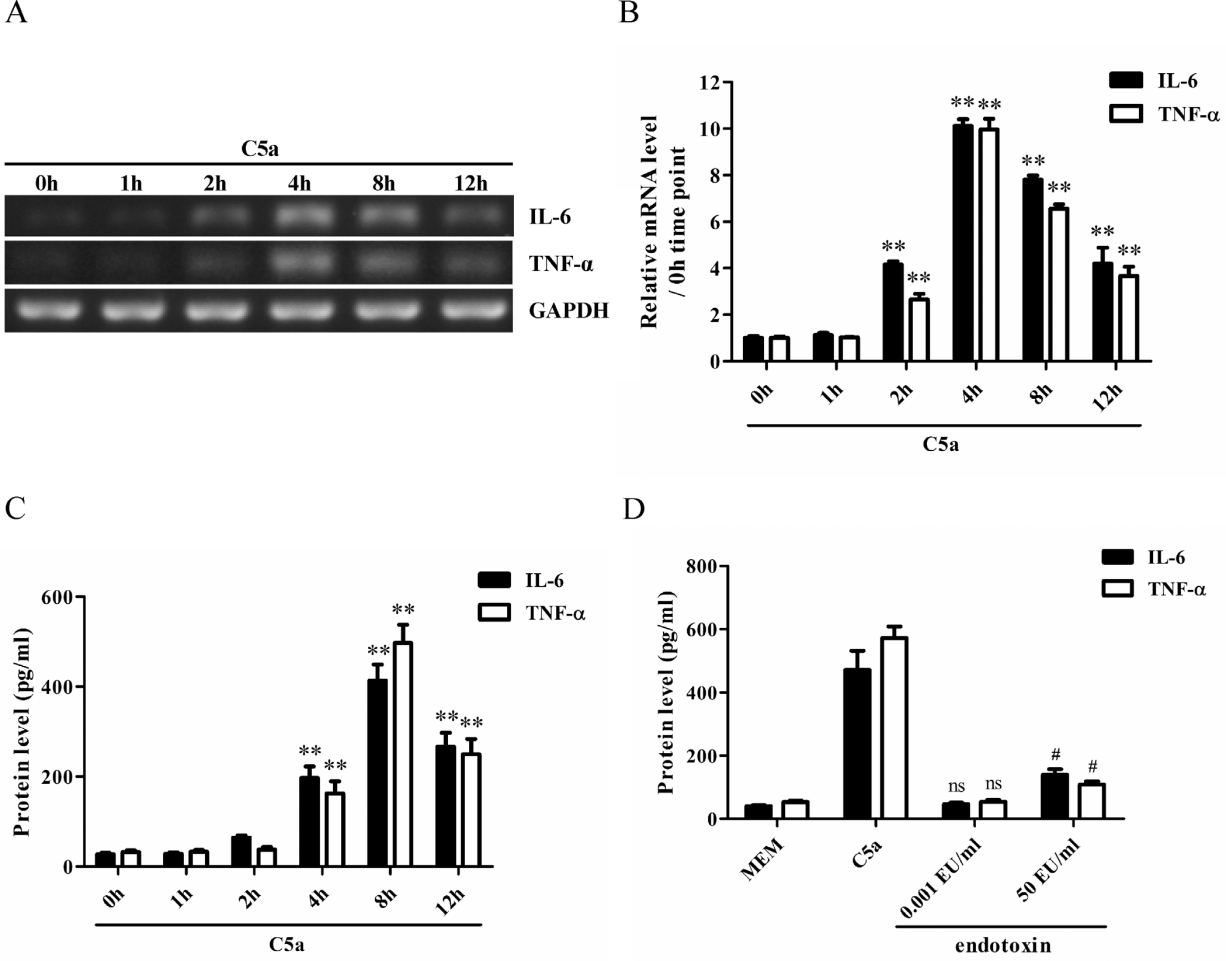
The expression of IL-6 and TNF-α in rat GMC stimulated with C5a. (A and B) RT-PCR analysis was performed to measure the mRNA levels of IL-6 and TNF-α in rat GMC exposed to C5a (50 ng/ml) for different time points (0 h, 1 h, 2 h, 4 h, 8 h and 12 h). (C) ELISA assay was done to detect IL-6 and TNF-α release from the GMC upon C5a stimulation (50 ng/ml) for above-mentioned time points. (D) ELISA experiments were performed to detect IL-6 and TNF-α release from the GMC stimulated with C5a at the dose of 50 ng/ml or with endotoxin at the dose of 0.001 EU/ml or 50 EU/ml for 8 h. ** *P*<0.01 *versus* 0 h time point (non-treated), ^ns^ *P*>0.05 *versus* MEM group, ^#^ *P*<0.05 *versus* MEM group. Results were represented as means ± SD (*n* = 3 in each time point). Representative photographs were displayed.

**Fig 4:**
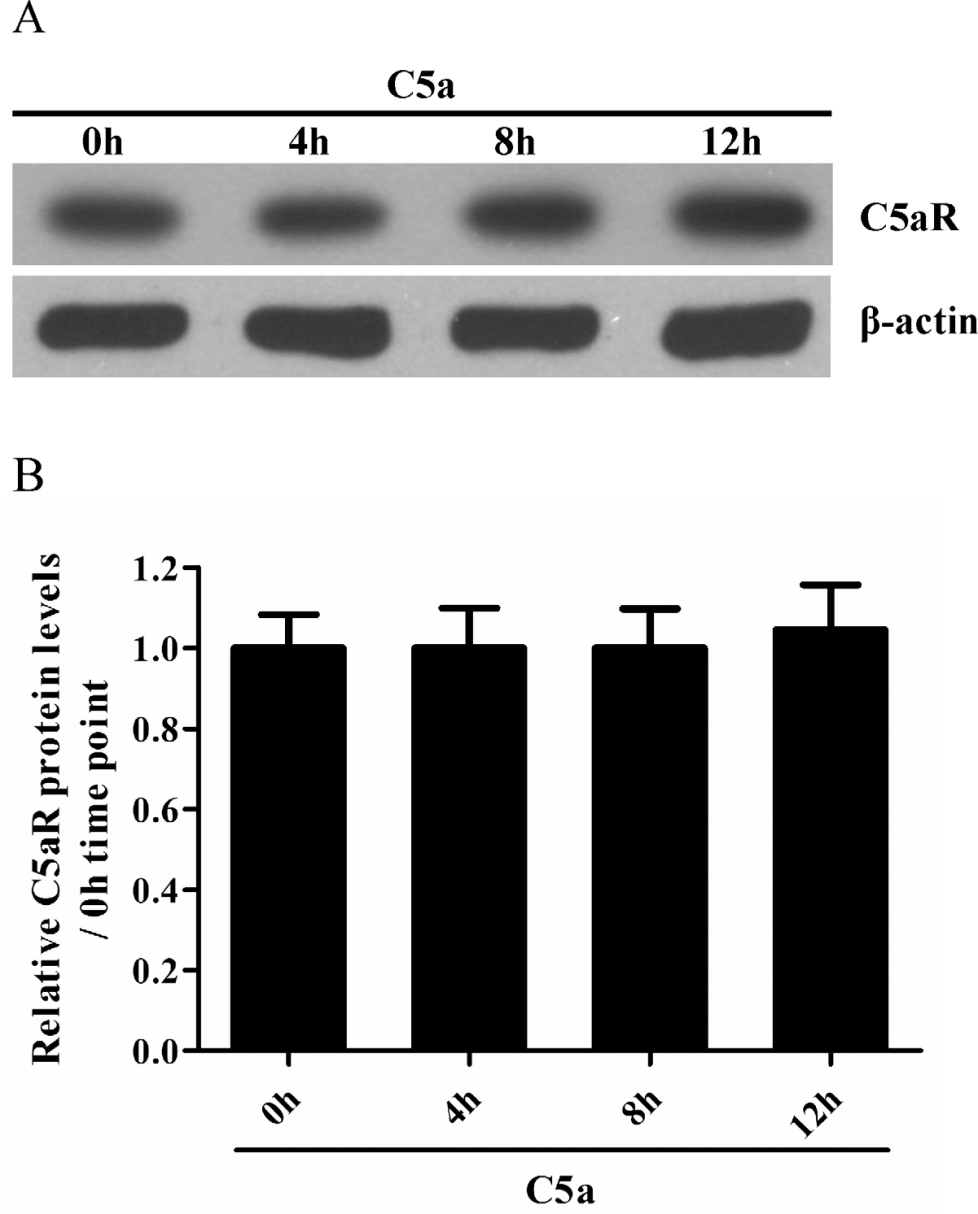
C5aR expression on rat GMC stimulated with C5a. (A and B) Western blot assay was performed to determine the expression of C5aR on the GMC incubated with C5a (50 ng/ml) for different time points (0 h, 4 h, 8 h and 12 h). Results were represented as means ± SD (*n* = 3 in each time point), and representative photographs were exhibited.

In order to determine whether C5a could induce the expression of IL-6 and TNF-α in rat GMC *in vitro*, the mRNA levels of IL-6 and TNF-α were measured by RT-PCR in the cultured rat GMC exposed to C5a for different time points (0 h, 1 h, 2 h, 4 h, 8 h and 12 h). The results showed that the mRNA level of IL-6 and TNF-α in the GMC began to increase at 2 h, peaked at 4 h after C5a incubation (Fig 3A and 3B). ELISA assay further showed that the release of IL-6 and TNF-α from the GMC was elevated at 4 h, and more obviously at 8 h after C5a stimulation (Fig 3C). Given that C5a protein prepared by prokaryotic expression could have been contaminated by endotoxin from Escherichia coli, in order to confirm that above-mentioned effects are caused by C5a rather than endotoxin, the level of endotoxin in C5a protein and its roles were determined. The result showed that the level of endotoxin in C5a stock solution was 10 EU/ml. That meant the level of endotoxin in C5a working solution (1:10000 dilution) was 0.001 EU/ml. Subsequent experiments exhibited that 0.001 EU/ml of endotoxin did not enhance the production of IL-6 and TNF-α in rat GMC, although a higher concentration of endotoxin (50 EU/ml) could increase the production of IL-6 and TNF-α (Fig 3D), indicating the up-regulation of IL-6 and TNF-α in rat GMC is indeed induced by purified C5a. Additionally, since IL-6 has recently been reported to up-regulate or down-regulate the expression of C5aR [34, 35], further experiment was designed to determine whether the expression of C5aR on the GMC was changed. The result displayed that there was not significant change of C5aR expression on the GMC membrane after C5a-stimulated IL-6 release (Fig 4). Collectively, these data indicate that C5a is able to induce the synthesis of IL-6 and TNF-α in rat GMC.

### p38 MAPK, ERK1/2 and JNK were activated in rat GMC in response to C5a

**Fig 5:**
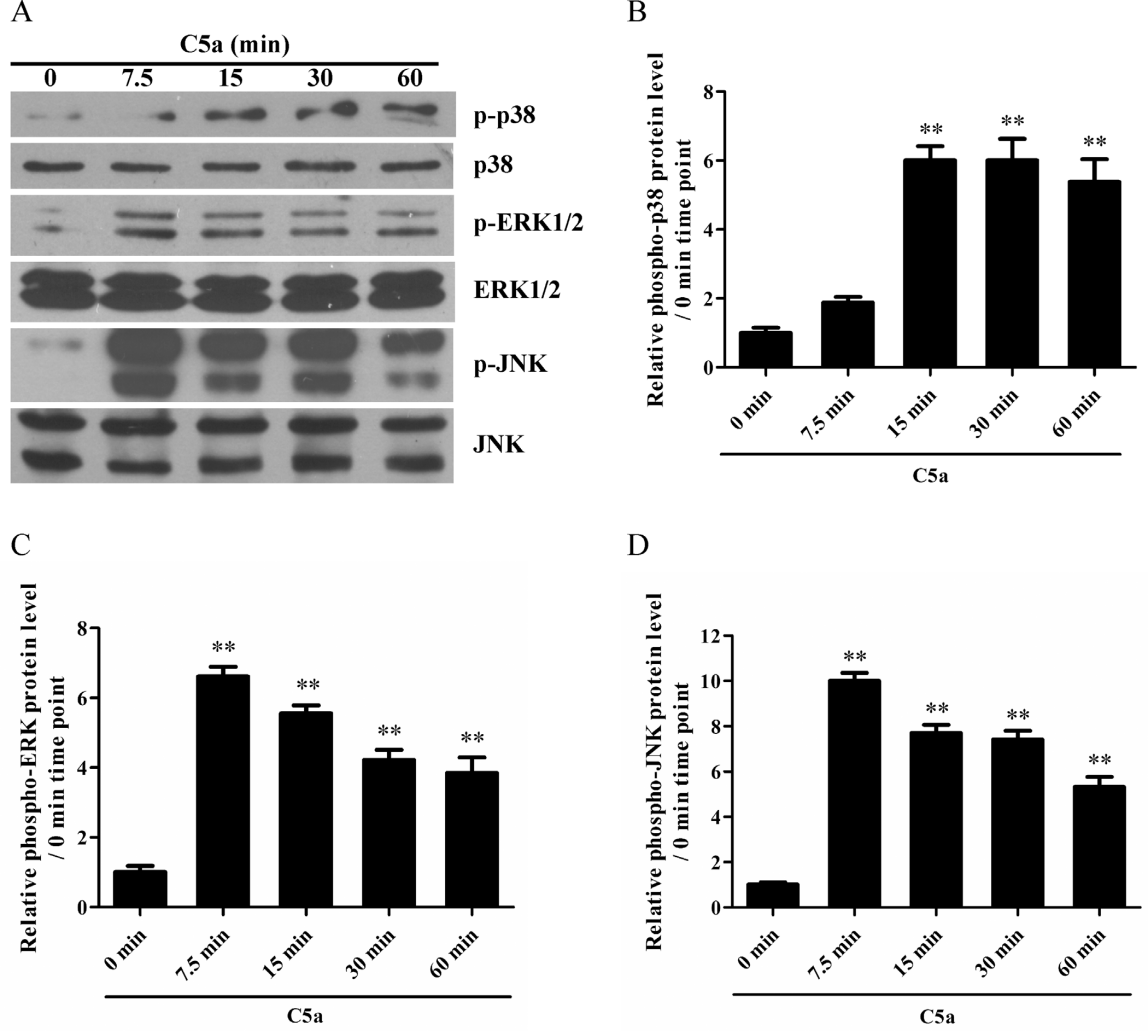
The phosphorylation of p38 MAPK, ERK1/2 and JNK in rat GMC treated with C5a. Western blot was done to detect the phosphorylation levels of p38 MAPK (A and B), ERK1/2 (A and C) and JNK (A and D) in the GMC exposed to C5a (50 ng/ml) for different time points (0 min, 7.5 min, 15 min, 30 min and 60 min). Results were represented as means ± SD (*n* = 3 in each time point). Representative photographs were manifested. ** *P*<0.01 *versus* 0 min time point (non-treated).

To explore the potential MAPK signal pathways involved in C5a-induced IL-6 and TNF-α production in the GMC, the phosphorylation levels of p38 MAPK, ERK1/2 and JNK were detected in the GMC exposed to C5a for different time points (0 min, 7.5 min, 15 min, 30 min and 60 min). Western blot analysis showed that C5a stimulation notably increased the phosphorylation levels of p38 MAPK, ERK1/2 and JNK in the GMC in a time-dependent manner with maximum increase at 7.5 min for both ERK1/2 and JNK or with maximum increase at 15 min and 30 min for p38 MAPK (Fig 5). These findings suggest that all of the above-mentioned three MAPK signaling pathways might be involved in C5a-induced IL-6 and TNF-α production in rat GMC.

### C5aR knockdown reduces the phosphorylation of p38 MAPK, ERK1/2 and JNK as well as the expression of IL-6 and TNF-α in C5a-induced GMC

**Fig 6:**
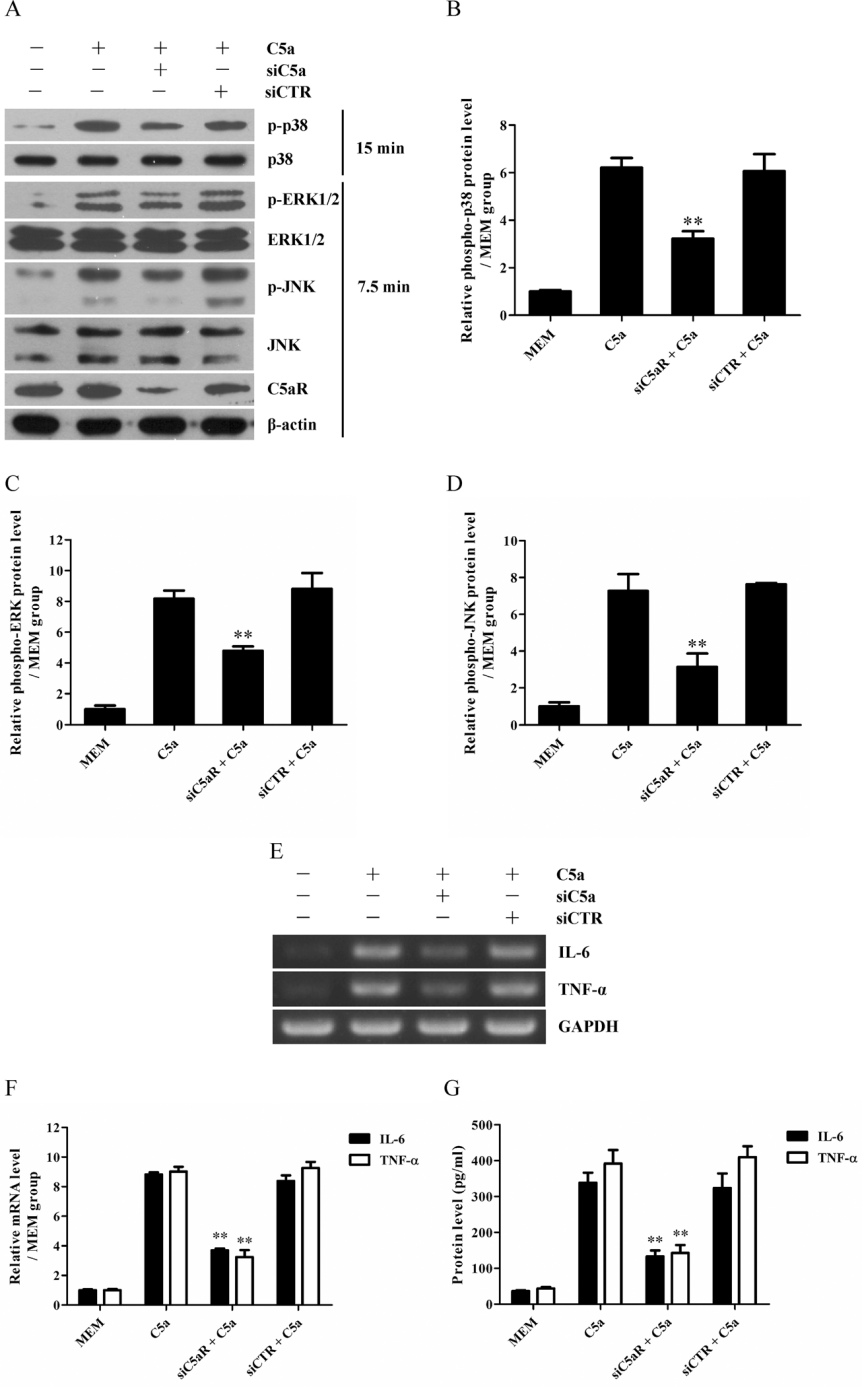
The effect of C5aR knockdown on the phosphorylation of p38 MAPK, ERK1/2 and JNK as well as the expression of IL-6 and TNF-α in C5a-induced GMC. siC5aR was transfected into the GMC to silence C5aR gene followed by C5a stimulation at the dose of 50 ng/ml for different time points, and then above-mentioned molecules were examined by Western blot, RT-PCR and ELISA respectively. (A-D) Western blot was performed to detect the expression of C5aR and the phosphorylation levels of p38 MAPK (A and B), ERK1/2 (A and C) and JNK (A and D) in the GMC after C5a stimulation for 7.5 min (C and D) or 15 min (B). (E and F) RT-PCR was done to determine the mRNA levels of IL-6 and TNF-α in the GMC after C5a stimulation for 4 h. (G) ELISA was used to detect the release of IL-6 and TNF-α from the GMC after C5a stimulation for 8 h. Results were represented as means ± SD (*n* = 3 in each group). Representative photographs were shown. ** *P*<0.01 *versus* C5a group and siCTR + C5a group.

In order to make sure C5a could increase the phosphorylation of p38 MAPK, ERK1/2 and JNK as well as the expression of IL-6 and TNF-α in rat GMC though its receptor C5aR, siC5aR was transfected into the GMC to silence C5aR gene before C5a stimulation, and then above-mentioned molecules were detected by RT-PCR, Western blot and ELISA respectively. The results showed that C5aR knockdown (Fig 6A) could not only effectively reduce the phosphorylation of p38 MAPK, ERK1/2 and JNK in the GMC at 7.5 min and 15 min after C5a stimulation (Fig 6A–6D), but also obviously suppress the production and release of IL-6 and TNF-α in the GMC at 4 h and 8 h after C5a stimulation (Fig 6E–6G), indicating that C5a induces the phosphorylation of p38 MAPK, ERK1/2 and JNK as well as the expression of IL-6 and TNF-α in the GMC through C5aR.

### The production of IL-6 and TNF-α was mediated differently by p38 MAPK, ERK1/2 and JNK signaling pathways in rat GMC stimulated by C5a

**Fig 7:**
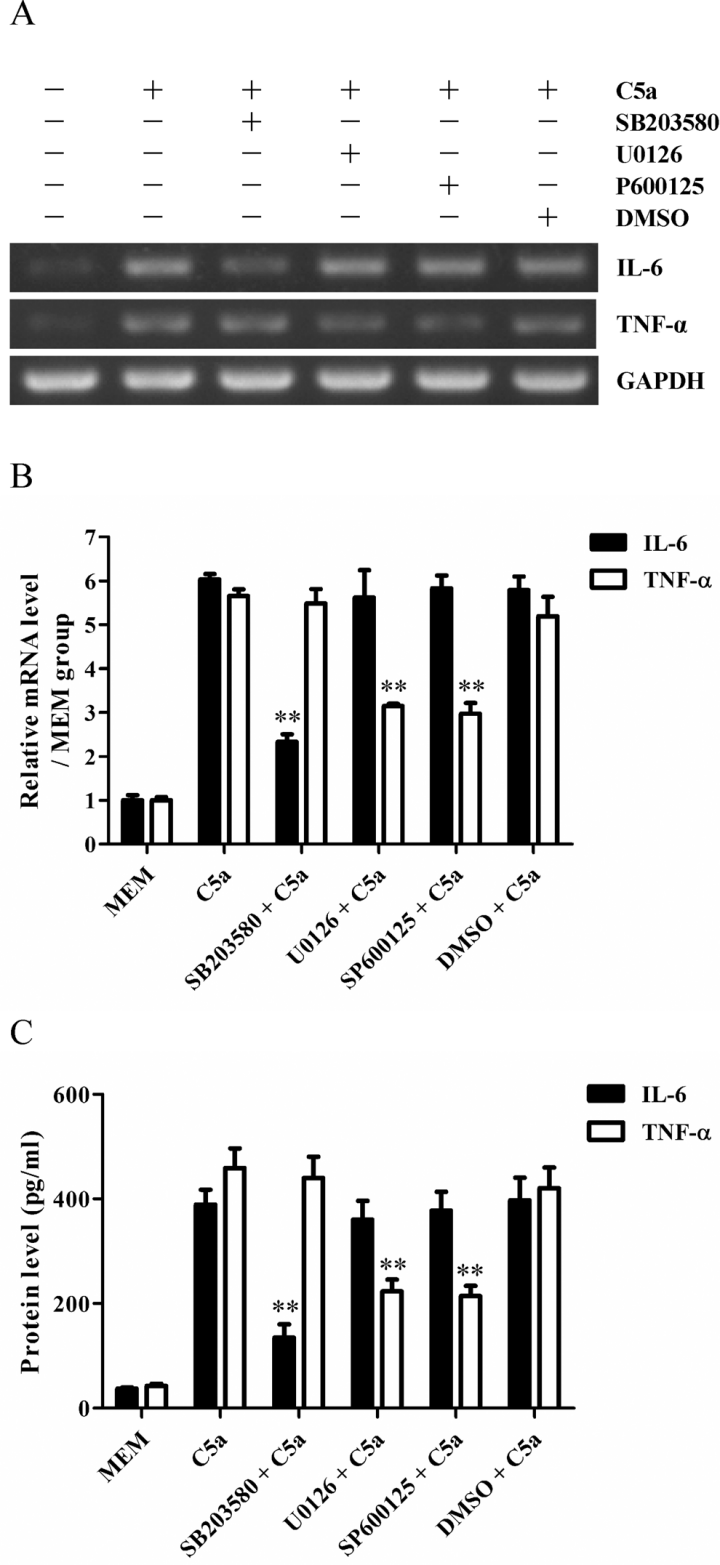
The roles of p38 MAPK, ERK1/2 and JNK in the production of IL-6 and TNF-α in the GMC stimulated with C5a. GMC were incubated with p38 MAPK inhibitor (SB203580, 10 μM), ERK1/2 inhibitor (U0126, 10 μM) and JNK inhibitor (SP600125, 10 μM) respectively for 30 min, and then stimulated with 50 ng/ml C5a for 4 h or 8 h. (A and B) RT-PCR was done to examine the mRNA levels of IL-6 and TNF-α in the GMC at 4 h. (C) ELISA was used to determine the release of IL-6 and TNF-α from the GMC at 8 h. Results were represented as means ± SD (*n* = 3 in each group). Representative photographs were exhibited. ** *P*<0.01 *versus* C5a group and DMSO + C5a group.

In this study, we have revealed that p38 MAPK, ERK1/2 and JNK signaling pathways were activated in rat GMC in response to C5a. In order to determine the roles of these signaling pathways in the production of IL-6 and TNF-α in the GMC exposed to C5a, p38 MAPK inhibitor (SB203580), ERK1/2 inhibitor (U0126) and JNK inhibitor (SP600125) were used respectively to inhibit the corresponding signaling pathways in the GMC before C5a incubation. Subsequently, the mRNA (at 4 h) and protein (at 8 h) levels of IL-6 and TNF-α were examined in the GMC stimulated with C5a by using RT-PCR and ELISA respectively. The results displayed that the level of IL-6 was remarkably decreased by p38 MAPK inhibitor, while the level of TNF-α was obviously decreased by either ERK1/2 inhibitor or JNK inhibitor (Fig 7), implying that p38 MAPK activation promotes IL-6 production, while either ERK1/2 or JNK activation enhances TNF-α production in the GMC with exposure to C5a.

## Discussion

It has been reported that inflammatory response plays important pathogenic roles in rat Thy-1N as an animal model of human MsPGN [14, 17, 18]. For example, the expression of pro-inflammatory cytokines including IL-6 and TNF-α is obviously up-regulated in the renal tissues and related to the pathological changes of rat Thy-1N [18, 24]. Our current studies found that the production of C5a, IL-6 and TNF-α was markedly increased in the renal tissues of rats with Thy-1N. More importantly, C5a production was earlier than IL-6 and TNF-α induction. Given that C5a is able to induce the synthesis of IL-6 and TNF-α in many other cells [26, 36–40], we hypothesized that C5a could promote the synthesis of IL-6 and TNF-α in the GMC. Notably, because there was no commercial rat C5a protein, in order to demonstrate our hypothesis, we constructed the plasmid of pET-21a/His-C5a successfully and produced recombinant rat C5a protein through prokaryotic expression. Subsequently, molecular weight of the recombinant rat C5a protein was identified by Western blot. The result showed that the molecular weight was about 11 KD that was very close to the predicted molecular weight (9.81 KD). Further experiments demonstrated that the recombinant rat C5a protein had the biological activities to induce ROS production and neutrophil chemotaxis, indicating that the recombinant rat C5a protein could be used to study the role of C5a in triggering the production of pro-inflammatory cytokines in rat GMC *in vitro*.

After the rat C5a protein with biological activities was prepared successfully, subsequent experiments were performed to determine the ability of rat C5a to induce the synthesis of IL-6 and TNF-α in rat GMC *in vitro*. The results showed that C5a could markedly up-regulate the expression of IL-6 and TNF-α in the GMC, and C5aR knockdown could greatly reduce the levels of IL-6 and TNF-α in the GMC stimulated with C5a, indicating that C5a could elevate the synthesis of IL-6 and TNF-α in the GMC though C5aR. Since IL-6 has recently been proven to be an important regulator of the C5aR [34, 35], next experiment was designed to confirm whether the expression of C5aR was effected on the surface of GMC. The result displayed that there was not significant change of C5aR expression on the GMC membrane after C5a-induced IL-6 production.

Reportedly, C5a can trigger MAPK activation in some target cells [41–43] and MAPK signal pathways are involved in mediating cellular IL-6 and TNF-α production [41, 44–47]. As a result, the regulatory roles of MAPK signaling pathways in C5a-induced IL-6 and TNF-α expression in the GMC were further explored in the present studies. The results displayed that C5a stimulation increased the phosphorylation levels of p38 MAPK, ERK1/2 and JNK in the GMC. Further analysis revealed that p38 MAPK activation regulated IL-6 production, while both ERK1/2 and JNK activation regulated TNF-α induction in the GMC exposed to C5a. Collectively, these findings suggest that MAPK signal pathways play crucial roles in the up-regulation of IL-6 and TNF-α in C5a-induced GMC.

It is worthy to mention that our previous study found that sublytic C5b-9 stimulation *in vitro* could induce the production of pro-inflammatory cytokines such as IL-6 [18] and TNF-α (data not shown) in rat GMC, while in the current studies, we revealed that C5a stimulation could also induce the synthesis of IL-6 and TNF-α in rat GMC. Taken together, these data indicate that complement activation products such as C5a and C5b-9 might cooperate to trigger the synthesis of pro-inflammatory cytokines in the GMC of rats with Thy-1N.

In summary, our present studies revealed that the levels of C5a, IL-6 and TNF-α were elevated in the renal tissues of rats with Thy-1N. Subsequently, recombinant rat C5a protein was produced and identified. Furthermore, rat GMC stimulated with our recombinant rat C5a *in vitro* obviously increased the expression of IL-6 and TNF-α, and the activation of MAPK signaling pathways was involved in mediating the synthesis of IL-6 and TNF-α. Specifically, p38 MAPK activation up-regulated IL-6 production, while either ERK1/2 or JNK activation up-regulated TNF-α production in the GMC exposed to C5a.
